# Characteristics of microRNAs and Target Genes in Maize Root under Drought Stress

**DOI:** 10.3390/ijms23094968

**Published:** 2022-04-29

**Authors:** Qi Tang, Haozhe Lv, Qimeng Li, Xiaoyue Zhang, Le Li, Jie Xu, Fengkai Wu, Qingjun Wang, Xuanjun Feng, Yanli Lu

**Affiliations:** 1 State Key Laboratory of Crop Gene Exploration and Utilization in Southwest China, Sichuan Agricultural University, Chengdu 611130, China; qitang927@gmail.com (Q.T.); lhzsicau@126.com (H.L.); lqm1892022@163.com (Q.L.); xiaoyuez0301@163.com (X.Z.); lile168170@163.com (L.L.); jiexu28@gmail.com (J.X.); wfk0909@163.com (F.W.); wdqdjm@126.com (Q.W.); xuanjun_feng@163.com (X.F.); 2 Maize Research Institute, Sichuan Agricultural University, Chengdu 611130, China; 3 Key Laboratory of Biology and Genetic Improvement of Maize in Southwest Region, Ministry of Agriculture, Chengdu 611130, China

**Keywords:** miRNA, drought stress, maize, root, target genes

## Abstract

Maize (*Zea mays*) is an important multi-functional crop. The growth and yield of maize are severely affected by drought stress. Previous studies have shown that microRNAs (miRNAs) in maize play important roles in response to abiotic stress; however, their roles in response to drought stress in maize roots is unclear. In our study, we found 375 miRNAs in the roots of 16 inbred lines. Of the 16 lines, zma-MIR168, zma-MIR156, and zma-MIR166 were highly expressed, whereas zma-MIR399, zma-MIR2218, and zma-MIR2275 exhibited low expression levels. The expression patterns of miRNA in parental lines and their derived RILs are different. Over 50% of miRNAs exhibited a lower expression in recombinant inbred lines than in parents. The expression of 50 miRNAs was significantly altered under water stress (WS) in at least three inbred lines, and the expression of miRNAs in drought-tolerant lines changed markedly. To better understand the reasons for miRNA response to drought, the degree of histone modifications for miRNA genes was estimated. The methylation level of H3K4 and H3K9 in miRNA precursor regions changed more noticeably after WS, but no such phenomenon was seen for DNA methylation and m6A modification. After the prediction of miRNA targets using psRNATarget and psRobot, we used correlation analysis and qRT-PCR to further investigate the relationship between miRNAs and target genes. We found that 87 miRNA–target pairs were significantly negatively correlated. In addition, a weighted gene co-expression network analysis using miRNAs, as well as their predicted targets, was conducted to reveal that miR159, miR394, and miR319 may be related to maize root growth. The results demonstrated that miRNAs might play essential roles in the response to drought stress.

## 1. Introduction

MicroRNAs (miRNAs) are small, non-coding RNAs, approximately 19–24 nucleotides (nt) in length, that play an important role in the regulation of mRNA degradation, translation inhibition, and chromosome modification [1,2]. miRNAs recognize their targets through sequence complementarity, and most miRNA targets participate in the process of growth and development in plants [3,4,5,6].

With the development of technology, sequencing has become an important technique used in miRNA research, with advantages such as a low sample size, high throughput, high accuracy, and ease of operation. It can be used to construct small RNA differential expression profiles between different samples and to discover novel miRNAs. Several miRNAs are reportedly involved in drought responses in plants. In wheat, the expression of miR159, miR160, miR169, miR166, miR172, and miR395 is regulated by drought [7]. miR164 can regulate drought resistance by targeting NAC genes coding transcription factors in rice [8]. Moreover, the overexpression of miR169 enhances drought tolerance by reducing stomatal opening, leading to decreased leaf water loss in tomatoes [9]. miR168, miR528, and miR167 are involved in drought response by regulating mitogen-activated protein kinase (*MAPK*), peroxidase (*POD*), and phospholipase D (*PLD*) in maize [10].

Usually, miRNAs can downregulate the expression of target genes via mRNA cleavage and translational inhibition through strict or short complementarity between miRNA and target mRNA in eukaryotes [11,12]. In plants, miRNAs repress targets via mRNA cleavage with nearly perfect complementarity, but there is still substantial evidence implying that miRNAs can be involved in target translation inhibition by affecting the function of ribosomes [13,14,15,16].

This study compares the expression of miRNA in the roots of the drought-tolerant (DT) AC7643 and drought-sensitive (DS) maize inbred lines AC7729/TZSRW, as well as their derived 14 recombinant inbred lines (RIL), under both well water (WW) and water stress (WS) conditions, in order to explore drought-responsive miRNAs and reveal the pattern of miRNAs and target genes under drought stress and explore the possible functions of miRNAs.

## 2. Results

### 2.1. Identification of miRNAs in Maize Root

**Figure 1:**
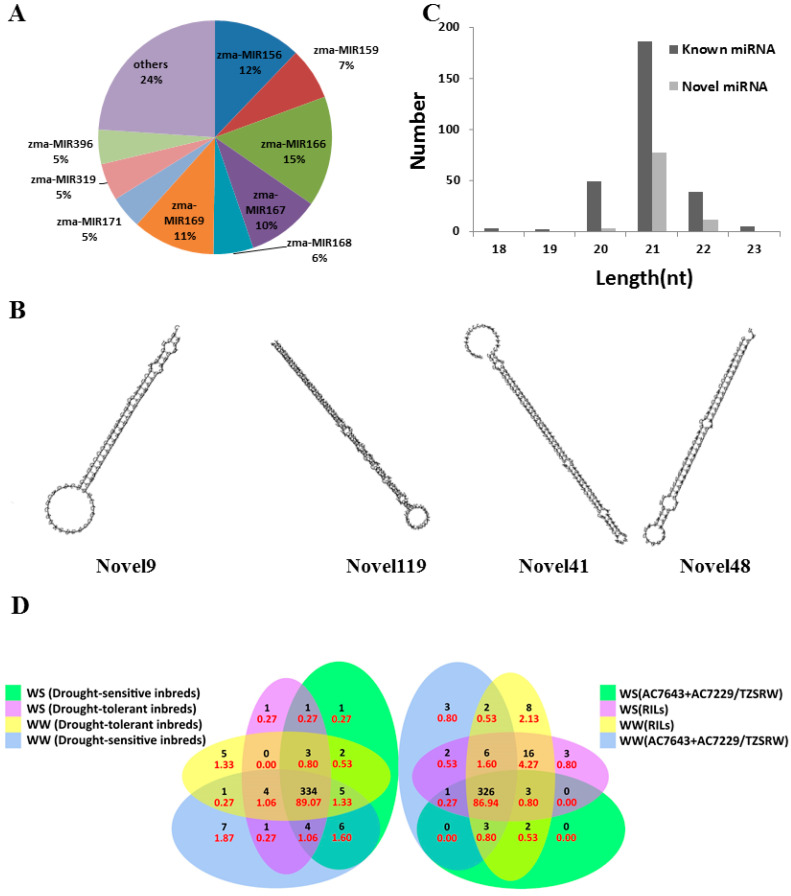
The composition and length distribution of miRNAs identified in maize root. (**A**) Composition of the identified known miRNAs. (**B**) Secondary structures of some novel miRNAs. (**C**) Length distribution of the identified miRNAs. Known, known miRNAs; Novel, novel miRNAs. (**D**) Venn diagram of parent miRNAs and RIL miRNAs in WW and WS (**right**); Venn diagram of drought-tolerant miRNAs and drought-sensitive miRNAs in WW and WS (**left**). The text in the Venn diagram depicts the proportion of miRNAs.

Following quality control processing, we obtained an average of 90,559,072 reads for each library from 32 maize root tissues. A total of 42.45% of clean reads were aligned to the maize v4 reference genome. We identified 284 known miRNAs from 28 miRNA families using the miRExpress and psRobot software. Among these, the zma-MIR169, zma-MIR395, zma-MIR171, and zma-MIR166 families accounted for approximately 10%, 10%, 9%, and 9%, respectively (Figure 1A). The reads that were not mapped to known maize miRNAs in miRBase 22 were used to predict novel miRNAs. Further, 91 novel miRNAs were identified in our materials (Supplementary File S1, Table S1), and their precursors were folded into the secondary hairpin structure by RNAfold (Novel9, Novel19, Novel41, and Novel48 are shown in Figure 1B). Of the identified miRNAs, 70.29% were 21 nt in length (Figure 1C). We compared the miRNAs of parents and RILs to determine whether there was a difference in RILs, and we detected more than 86% of miRNAs in both parents and RILs (Figure 1D). RILs were classified as drought-tolerant and drought-sensitive based on leaf mortality (Supplementary File S2, Figure S1). Most miRNAs were detected in both groups (Figure 1D).

### 2.2. Expression Pattern of Identified miRNAs in Maize Root

**Figure 2:**
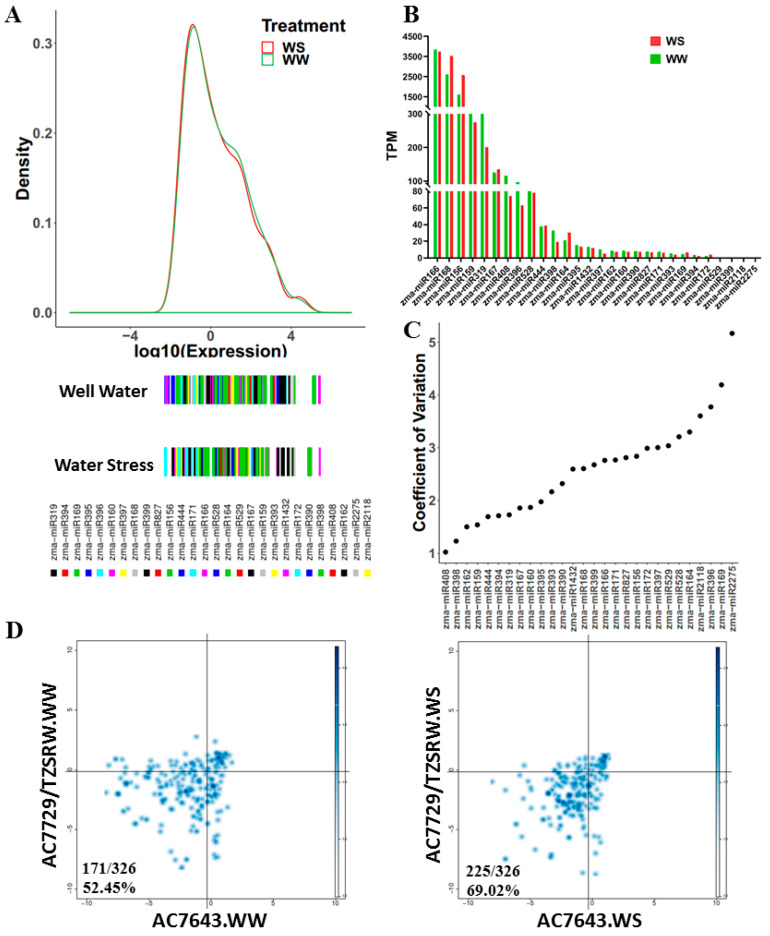
Expression analysis of miRNAs identified in maize root. (**A**) Distribution of identified miRNA expression in WW and WS. (**B**) Expression of miRNA families in WW and WS treatments. (**C**) Expression variation of miRNA families in 16 materials. (**D**) Expression of miRNAs between parents and RILs. We standardized the expression of miRNAs in 14 RILs. *X*-axis, values of the expression of RILs minus AC7643 (DT); *Y*-axis, values of the expression of RILs minus AC7729/TZSRW (DS).

The expression of miRNAs was described by the expression abundance (TPM = miRNA reads/total mapped reads × 10^6^). By analyzing the expression of miRNAs, the results revealed a wide expression distribution range between the identified miRNAs, and there was no significant difference in the expression of the identified miRNAs in WW and WS (*p* = 3.318 × 10^−1^ t-value = −9.7049 × 10^−1^, df = 15,267, *t*-test, Figure 2A). However, miRNAs in different families showed distinct differences in their expression patterns. miRNAs in the zma-MIR168, zma-MIR156, and zma-MIR166 families were highly expressed under the two treatments, and their TPMs were greater than 2500; meanwhile, the expression of family members from zma-MIR399, zma-MIR2218, and zma-MIR2275 was very low under the two treatments with TPMs of less than 10 (Figure 2B). These six miRNA families were specifically expressed, unlike zma-MIR408, zma-MIR398, and zma-MIR162, in 16 lines (Figure 2C). We compared the expression of miRNAs identified in both the parents and RILs. Interestingly, over 50% of the miRNAs tended to be down-regulated in RILs compared to the parents in the WW and WS treatments (Figure 2D). These results indicate the consistent expression patterns of miRNA families under WW and WS, but the expression of most miRNAs was lower in RILs than in parents.

### 2.3. Differential Expression of miRNAs under Drought Condition

**Figure 3:**
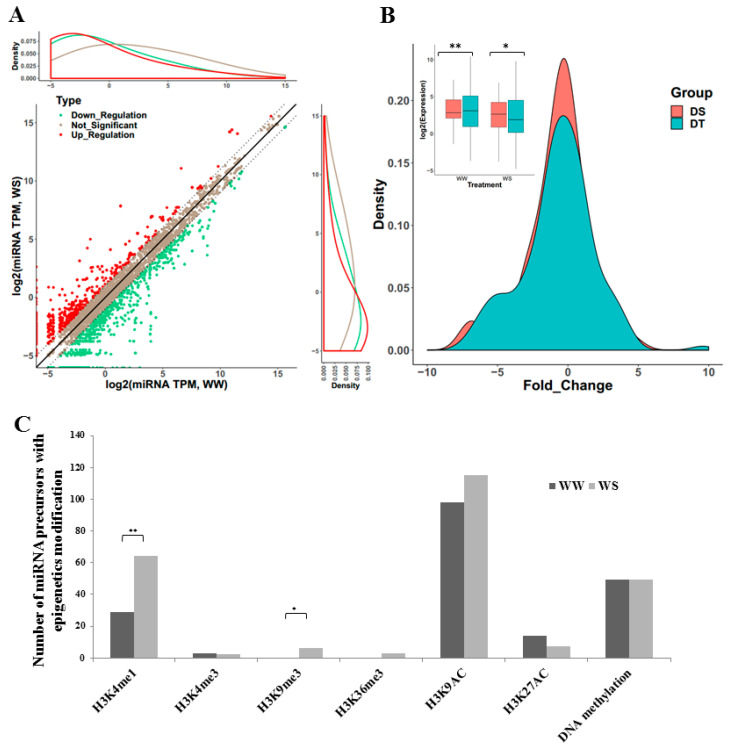
Differential expression of identified miRNAs in maize root. (**A**) Log2 fold change of miRNAs under WS. Down-Regulated means log2 fold change < −1; Up-Regulated means log2 fold change > 1; Not-Significant, −1≤ log2 fold change ≤ 1. (**B**) The difference in miRNA expression and log2 fold change between drought-tolerant and drought-sensitive lines. DT, drought-tolerant; DS, drought-sensitive. * for *p-*value ≤ 0.05, ** for *p-*value ≤ 0.01. (**C**) The number of miRNA precursors with epigenetics modification. Chi-square test, * for *p*-value ≤ 0.05, ** for *p*-value ≤ 0.01.

By comparing the expression of miRNAs between WW and WS, we detected 1097 up-regulated miRNAs (log2 fold change > 1) and 1547 down-regulated miRNAs (log2 fold change < −1) in 16 materials (Figure 3A). Using edgeR for differential expression analysis, we identified 20 significantly up-regulated miRNAs and 30 significantly down-regulated miRNAs in at least three lines (FDR ≤ 0.05).

Additionally, the expression of 20 miRNAs significantly changed in at least three materials after WS treatment in drought-tolerant materials and 17 miRNAs in the drought-sensitive group. Moreover, the expression of these two groups was significantly different in WW and WS (*p* = 4.727 × 10^−4^, t-value = 3.5674, df = 164.32, *p* =2.504 × 10^−2^, t-value = 2.2618, df = 162.12, *t-*test), and the expression in drought-tolerant material changed more strongly than that in drought-sensitive material after WS treatment (*p* = 8.27 × 10^−7^, D = 0.36364, Kolmogorov–Smirnov test, Figure 3B).

Normally, gene activity is controlled not only by DNA sequences but also by epigenetic modifications. Therefore, we searched for histone modifications (H3K4me1, H3K4me3, H3K9me3, H3K36me3, H3K9AC, and H3K27AC) and DNA methylation in the upstream or downstream 2 Kb of the miRNA precursors. The degree of histone modifications became stronger after WS treatment while the degree of DNA methylation was insensitive, especially for the degree of H3K4me1 and H3K9me3 (*p* = 2.84 × 10^−4^, X-squared = 13.172, df = 1, *p* = 1.431 × 10^−2^, X-squqred = 6, df = 1, χ^2^ test, Figure 3C). We further checked the m6A modification in the upstream or downstream 1 Kb of the miRNA precursors and found that only a few miRNA precursors (5.84%) had m6A modifications (Supplementary File S1, Table S2). These results suggest that histone modifications might regulate the expression of miRNA precursors after water stress.

### 2.4. The Characteristics of Target Genes Regulated by miRNAs

**Figure 4:**
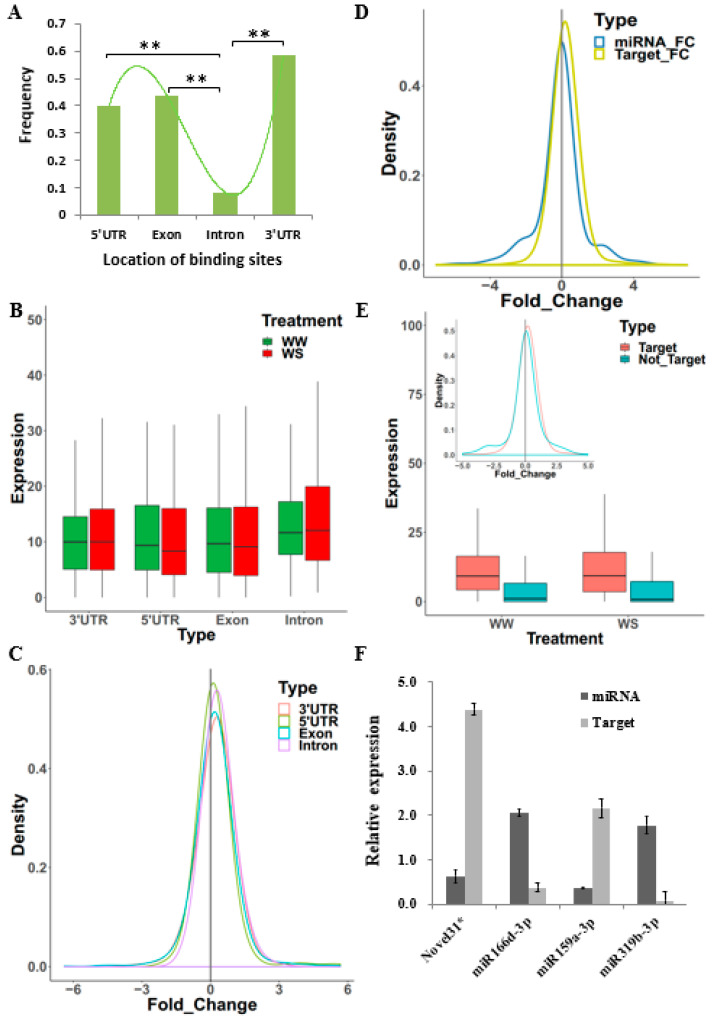
The analysis of negatively correlated miRNA/target pairs. (**A**) The location of miRNA target sites on targets. Chi-square test, ** for *p-*value ≤ 0.01. (**B**,**C**) The target expression and log2 fold change of different miRNA binding site. (**D**) Fold change distribution of negatively correlated miRNA/target pairs. (**E**) Comparison of expression and log2 fold change between target genes and other genes. Target, miRNA target genes; Not_Target, the genes were not miRNA target genes. (**F**) The real-time PCR analysis of expression of miRNAs and targets in AC7643. The expression of each gene in plants grown in WW was set to 1. *Y*-axis means the relative expression of each gene in plants grown in WS. Data are shown as mean ± SD (*n* = 3) of three independent biological replicates. From left to right, the target genes are Zm00001d003518, Zm00001d048527, Zm00001d053589, and Zm00001d053545.

After miRNA target prediction, 604 miRNA–target pairs were detected. miRNAs typically repress their targets. We wanted to obtain more reliable miRNA–target pairs; therefore, the correlation between the expression of miRNAs and their corresponding targets in 16 materials was tested. Eighty-seven miRNA–target pairs were significantly negatively correlated, with an average correlation coefficient of −0.48 (*p* ≤ 0.05, Spearman correlation). We regarded these pairs as reliable and focused on them in further analyses. We observed that the miRNA-binding sites on targets were enriched in untranslated regions (UTRs) and exon regions in our study (Figure 4A). Furthermore, we found that the expression of the target miRNA bound to UTRs and exon regions was lower than that bound to intron regions, and that the 5′UTR was significantly lower (*p* ≤ 0.01, *t-*test, Figure 4B). The distribution of log2 fold change was different among different regions, and there was a significant difference between the 5′ UTR regions and intron regions (*p* = 1.511× 10 ^−3^, D = 0.18099, Kolmogorov–Smirnov test, Figure 4C). From these results, it was understood that most miRNAs regulate the expression of target genes by binding to the untranslated region of the target gene.

In general, miRNAs tended to be down-regulated, while targets tended to be up-regulated in our study (Figure 4D). Additionally, the expression of target genes was significantly higher than that of non-target genes, and target genes changed more noticeably after WS (WW: *p* = 3.826 × 10^−^^4^, t-value = 3.5639, df = 1001.6, *t*-test, *p* = 8.882 × 10^−^^16^, D = 0.22678, Kolmogorov–Smirnov test, Figure 4E). Additionally, four miRNA–target pairs were randomly selected for qRT-PCR verification, and the details are shown in Supplementary File S1, Table S3. After water stress treatment, miRNAs and their targets showed opposite changes in AC7643 (Figure 4F). This further confirms our prediction of reliable miRNA targets.

### 2.5. Co-Expression Network Analysis of miRNAs and Targets

**Figure 5:**
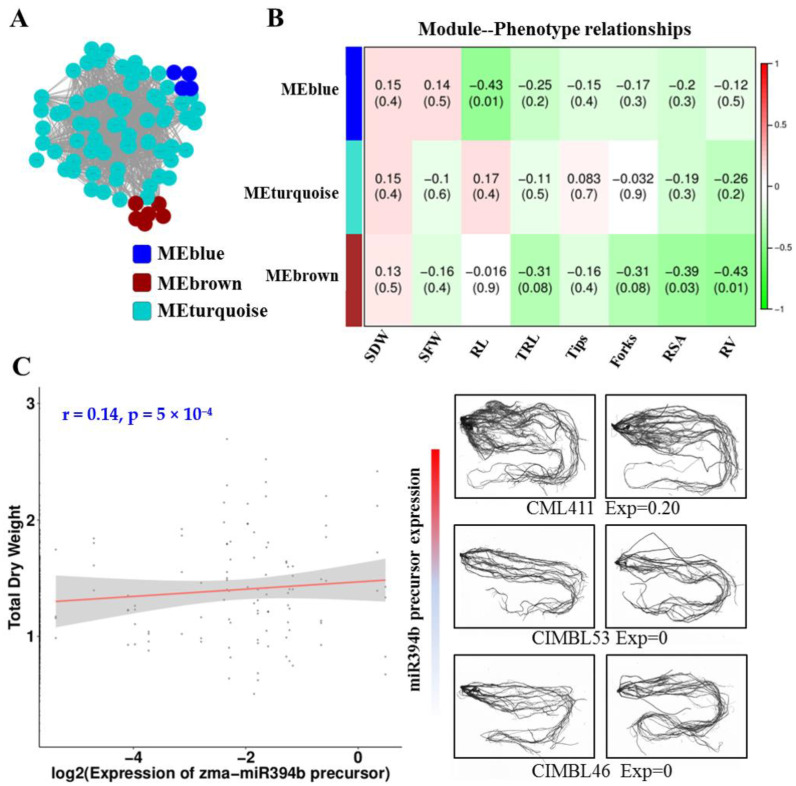
Co-expression network analysis in well water and water stress treatments. (**A**) Network visualization in Cytoscape. The nodes were colored by module membership. (**B**) Correlations between module eigengenes and root phenotypic traits. The numbers within the heatmap represent correlations and *p-*value (red, positively correlated; green, negatively correlated) for the module–trait associations (SDW, shoot dry weight; SFW, shoot fresh weight; RL, root length; TRL, total root length; Tips, root branches; Forks, root forks; RSA, root surface area; RV, root volume). (**C**) The connection between zma-miR394b precursor expression and total dry weight. On the left is the root phenotype of some lines from a natural group containing 368 lines. Red means that the expression of zma-miR394b precursor is higher (**right**).

To find more connections between miRNAs, their targets, and phenotypic variations, we applied weighted gene co-expression network analysis (WGCNA) to decipher their regulations. The TPM values of miRNAs and the FPKM values of targets in WW and WS were used to construct the co-expression network after variance-stabilizing and filtering. The network contained three modules, as shown in Cytoscape, with an edge threshold cutoff of 0.15 that retains about 85% miRNAs and targets (Figure 5A). To determine the relationship between the modules and the root phenotypic traits, we used WGCNA to correlate the module eigengenes with root phenotypic traits. There was a significant negative correlation between the MEblue module eigengenes and root length (*p* = 0.01, Figure 5B) that contained four members of zma-MIR159. Meanwhile, the MEbrown module was significantly negatively correlated with root surface area and root volume, with four zma-miR319 miRNAs and two zma-miR394 miRNAs (*p* = 0.03, 0.01, Figure 5B). In addition, zma-miR394b, with the largest relevance value in the MEbrown module, was significantly positively correlated, with a total dry weight in a natural group that contained 368 materials, and the root of the line with a higher expression of zma-miR394b precursor was stronger than the line with a lower expression of the same (*p* = 5 × 10^−^^4^, Figure 5C). This suggests that the miRNAs in the MEblue and MEbrown modules were related to maize root growth.

## 3. Discussion

In recent years, many studies have shown that miRNAs play important roles in the response to high salinity, temperature stress, and drought stress in plants [8,9,17,18]. Drought stress, an important type of abiotic stress, is effective in enhancing drought tolerance by increasing rooting and changing root architecture in plants [19,20,21]. Thus, further studies on the expression pattern of miRNAs in roots should help to enrich our knowledge of drought-tolerance mechanisms in maize. In our study, we identified 284 known miRNAs and 91 novel miRNAs in 16 materials under well water and water stress, and 70.29 of them were 21 nt in length, a typical length in plants [22]. Over 86% of miRNAs were expressed in both parents and RILs, and more than 50% of them were expressed at lower levels in RILs than in parents. In hybrids, most miRNAs tend to be down-regulated compared to the miRNA of their parents [23,24,25]. RILs produced by hybridization may also appear, meaning that most miRNAs tend to be down-regulated compared to their parents.

Comparing the expression of miRNAs between well water and water stress, we found 50 miRNAs that significantly responded to water stress, some of which were members of the miR156, miR160, and miR166 families. Previous research has revealed that miR156 can be involved in the drought response by regulating target *SPL* [26,27]. In addition, miR160 and miR166 both participate in root development by regulating their targets, indicating their relationship to drought response [28,29]. miRNA expression changed substantially more in drought-tolerant than in drought-sensitive plants under water stress. This indicates that these miRNAs, which significantly respond to water stress, are related to drought tolerance in maize seedlings. Histone modifications have been shown to regulate gene expression [30,31,32]. The degree of histone modification in 2 Kb of the miRNA precursors, upstream or downstream, became stronger than DNA or RNA methylation after WS. That suggests that histone modifications might regulate the expression of miRNA precursors under WS.

miRNAs usually repress their targets by mRNA degradation and translation inhibition, and there is a negative regulation between miRNAs and target genes. By correlating the expression of miRNAs and their target genes, we found 87 miRNA–target pairs that were significantly negatively correlated. In a recent study, except for binding to the 3′ UTR, AtAGO1-RISC can repress target expression by blocking the recruitment or movement of ribosomes by binding to the 5′ UTR or open reading frame (ORF) in *Arabidopsis* [16]. Our results were consistent with the observation that miRNA target sites were enriched on UTRs and ORFs, and qRT-PCR verification showed opposite changes between miRNAs and targets. Taken together, these results prove that our prediction of miRNA targets is reliable.

To speculate the function of miRNAs in maize roots, we applied WGCNA to generate a co-expression network associated with root phenotypic traits. A module containing four members of zma-MIR159 was significantly negatively correlated with root length. Another module containing four miRNAs from zma-miR319 and two miRNAs from zma-miR394 was significantly negatively correlated with root surface area and root volume. In *Arabidopsis thaliana*, it has been confirmed that miR159 is a negative regulator of primary root growth [33], and miR394a over-expressing plants display drought tolerance and are more sensitive to ABA treatment in root growth compared to such treatments in wild-type plants [34]. The overexpression of miR319 can enhance plant drought tolerance by improving water retention and cell membrane integrity in shoots [35]. As described previously, increasing rooting can strengthen the drought tolerance of plants. This suggests that the two modules may be involved in drought response through root growth regulation; miR159, miR319, and miR394 may affect root growth in maize. We confirmed this by performing additional molecular experiments and phenotypic identification.

## 4. Conclusions

The objective of this study was to explore the functions of miRNAs and determine the influence of drought stress on miRNAs and target genes in maize root. In our study, the miRNA expression was different among the miRNA families, and over 50% of miRNAs were expressed at lower levels in the recombinant inbred lines than in the parent lines in the two treatments. The degrees of H3K4 methylation and H3K9 methylation in miRNA precursors may help to regulate the expression of miRNA precursors after WS. After correlation analyses and the qRT-PCR verification of miRNAs and targets, we constructed a co-expression network and found that miR159, miR394, and miR319 are probably related to maize root growth. This study lays a foundation for further research on drought tolerance mechanisms in maize.

## 5. Materials and Methods

### 5.1. Plant Material and Treatment

The maize lines used in the study were as follows: the drought-tolerant inbred line AC7643; drought-sensitive inbred line AC7729/TZSRW; and their 14 RIL lines (RIL208, RIL165, RIL142, RIL203, RIL155, RIL131, RIL231, RIL64, RIL166, RIL8, RIL47, RIL27, RIL226, and RIL126). The lines used in this study were provided by the International Maize and Wheat Improvement Center (CIMMYT). The materials were grown in Hoagland’s solution culture, and the plants of each line were randomly divided into control and treatment groups. At the five-leaf stage, the water-stress group was treated with 20% (*w*/*v*) polyethylene glycol PEG 6000 (Sigma-Aldrich, Saint Louis, MO, USA) for 24 h. For each sample, at least three plants were pooled, and two independent biological replicates were used.

### 5.2. Library Construction and Sequencing

Total RNA was extracted from the whole roots using TRIzol^®^ (Invitrogen, USA), and treated with RNase-free DNase I. smRNA libraries were constructed from the extracted total RNA using commercially available reagent sets, following the instructions by the manufacturer (Solexa) and Wang et al. [36]. Afterwards, 16~35 nt smRNAs were separated from the total RNA using 15% denaturing PAGE (polyacrylamide gel electrophoresis) and ligated to adaptors at the 5′ and 3′ ends. Ligation products were gel-purified using 10% denaturing PAGE and reverse-transcribed. For replicates I and II, libraries were sequenced on HiSeq 2000 and HiSeq 2500 SE50 systems (Illumina, San Diego, CA, USA).

### 5.3. miRNA Identification and Data Processing

After quality filtering, the high-quality reads were aligned to the maize v4 reference genome and known miRNAs were identified using the miRExpress [37] and psRobot [38] software with default parameters. For novel miRNA identification, shortstacks [39] and miRCat [40] were used with default parameters, based on the criteria of Meyers for plant miRNA annotation [41], which requires the reads of miRNA/miRNA* to exceed 20 in at least three libraries or exceed three in at least ten libraries.

miRNA targets were predicted using psRNATarget [42] and psRobot without any bugle. For differential expression analysis, we used the Bioconductor package edgeR [43] in the statistical program R [44]. An exact test was conducted to detect miRNAs, with significant differences in expression found between WW and WS (FDR ≤ 0.05).

### 5.4. RNA Analysis

For qRT-PCR, RNA reverse transcription was performed using the PrimerScript^TM^ RT reagent Kit with the gDNA Eraser (TAKARA, Shiga, Japan). For miRNAs, we used stem-loop qRT-PCR, as previously described by Chen et al. [45]. ChamQ^TM^ Universal SYBR qPCR Master Mix (Vazyme, Nanjing, China) was used for real-time PCR.

### 5.5. WGCNA Network Analysis

The data for co-expression network analysis were filtered to remove genes that did not reach an FPKM or TPM value of one in at least three materials. Then, the Log2-normalized FPKM and TPM values were used to generate the network by WGCNA [46]. Module eigengenes were used for the network to relate the root phenotypic traits.

## Data Availability

The raw sequencing data of 16 lines were deposited in the SRA database (accession number: PRJNA294848, PRJNA816639).

## Supplementary Materials

The following supporting information can be downloaded at: https://www.mdpi.com/article/10.3390/ijms23094968/s1.
