# Supplementary material for: Characteristics of microRNAs and Target Genes in Maize Root under Drought Stress
Source: Int J Mol Sci. 2022 Apr 29;23(9):4968. doi: 10.3390/ijms23094968 (PMC9104622; doi:10.3390/ijms23094968)
Supplement: Supplementary file 1 [file ijms-23-04968-s001.zip › ijms-1682091-supplementary.pdf]

Supplement

Table S1. The detail of the novel miRNAs.

ID	chromosome	start	end	strand	Precursor_seqence	miRNA_seqence	miRNA*_seqence
Novel1	10	143745135	143745221	-	GAUAUUGGCGCGGUUCAAUCAGAAAGCUUGCGCUCCAGGCCCGAGGGGCUCCACUCUUUGAUUGAGCCGUGCCAAUAUC	UGAUUGAGCCGUGCCAAUAUC	
Novel2	1	114053928	114054053	-	UGCAAGGUGAGAGAAUGGUAUAAUCACAAUUAACGCUGAAAAUAUUGUUGAAAUUUUCAAGUUUAAUUGUGAUUAUACUCUUCUC UCACAUUGCAAUUGUGAUGUUACCCC	UGAGAGAAUGGUAUAAUCACA	UGAUUAUACUCUUCUCUCACA
Novel3	1	118284899	118284973	+	CCCGCCGGCGAGCGCUUUCCUGUGAGGGCGCCGCCGUCGGUGAG	GUGAGGGCGCCGCCGUCGGUGA	
Novel4	1	118402446	118402582	+	GCCAGAUUCCUCGUCCCUUCUGUUCACCGGCCAGGCGGAGGACUGAUCCGUCGUCCAUUGUUGCGCUGCCGUCGAACUCCCCGCC GGCGAGCGCUUUCCUGUGAGGGCGCCGCCGUCGGUGAG	GUGAGGGCGCCGCCGUCGGUGA	
Novel5	1	182818341	182818466	-	AGGACUAGUUUGGGAGCUAAAAUACCGGAGGGGAUUGGAGAGAUUAAAAUCCCCUUAUUUAAAAUUGAAUAAGAAAGGGAUUUUA GCCCCUCCAAUCCCCUCCGGUAUUGUGGUUCCCAAACUAGC	UACCGGAGGGGAUUGGAGAGA	CCUCCAAUCCCCUCCGGUAUU
Novel6	1	244623818	244623928	-	GCAUCCAAUCAAAAUCUAACAAUUACUGCUAUUUUAGGUGGAAGAGGUUAAAUGUAAAAUAGACAUUAAGUUUCUGGUGCAGUCA CCGUUGGAUUUUGAUUGGAUGCAC	UUGGAUUUUGAUUGGAUGCAC	GCAUCCAAUCAAAAUCUAACA
Novel7	1	248444551	248444633	+	UUAUUCCCUAAAUUGCCAAACACAAGGACUAAAAUAGAGAUAAUUCCUCUUUUAAUCCAUGUGUUUGGCAAUUUAGGGACUAA	AUUCCCUAAAUUGCCAAACAC	GUUUGGCAAUUUAGGGACUAA
Novel8	1	303789472	303789551	+	CUCUGAUAGCCAAGCAUGAUUUGCCCGUAGUCUGACUCUGAGUGUGCUCCUGCUACGGGCAAAUCAUCUUGGCCAUCUGA	UAGCCAAGCAUGAUUUGCCCG	GGCAAAUCAUCUUGGCCAUC
Novel9	1	32605445	32605565	-	UUGUUUGGGAACAAGGAUACUAGAGGGGAUUGGAGGGGCUAAAAUCCCCUUGCUUUUCAAAUCUGAAUAGCAAGAAAAUUUUAGC ACCUCCAAUCCCUUCCGGUAUUCUUGCUC	AGCACCUCCAAUCCCUUCCGG	AGAGGGGAUUGGAGGGGCUAA

ID	chromosome	start	end	strand	Precursor_seqence	miRNA_seqence	miRNA*_seqence
Novel10	2	10174728	10174866	-	UCCACCCCAUCAAUCUUAAAGUAAUAGAACACCGAUCUUAAAGCAAUAGAACAGCCUGCAGAGAAGUGGCUCGGUGUUCUGUUGCUUUAAGAUUGAUGGGGUGGACAUG	GCUUUAAGAUUGAUGGGGUGG	ACCCCAUCAAUCUUAAAGUAA
Novel11	2	198436177	198436331	+	CUGGCAUGGCGAUGGAAGCUCUGCUUCGGUAGCCAAGGAUGAGCUGCCUGUGGCCUCCUGCUGCGGACGUUGCGUGGCCCCGCCUCCACCGCGUGCGGUCCCCGCAGGCAGCCUCCUUGGCUAGUCUGAGCGGCUUCCAUCCUCCAUGCUAGGCU	UAGCCAAGGAUGAGCUGCCUG	
Novel12	2	32718029	32718291	-	AACAGGUCCUAAAACUUGGUUCGGCCGUGCAAAACCAUAAAAAAUCAGGUUUGCUCAAACCGGAUGCUAACGUGGUGUGCCAUGUUGGAACACACUAAUGAAAGUUCGGGGACCCAAAUGACAUAUUAUACCAACUUAAGAGACUCAAGUGACACAGCAAUGCCAAGUUCUAGGACCCAAGUGAGUCUAACGUGGCACGCCACGUCGGUAUCCGGUUUGAGCAAACCUGGUUUUAGAUGGGCCGCCAAGUUCCAACACAUGUU	ACGCCACGUCGGUAUCCGGUU	CCGGAUGCUAACGUGGUGUGC
Novel13	2	92156518	92156670	+	UUAUGCAGAACAAUUUACAGACGGAAAUGAUUUGUACUAUUUCUCAGGUGCAAACCAACCAUUUCUCAGGUGCAAACCAUUUCGAUCUGUAAGUUGUUGUGCACAAGAAUACUACUGUUUUAUUUUAUAUUUUGGAAACUGCUGUUUGGAUAA	AUGCAGAACAAUUUACAGACG	UCUGUAAGUUGUUGUGCACAA
Novel14	3	123760321	123760453	-	AAGGGCUGAUUUGUUGAUCGGGGAUCCCGAGGGGAUCCAUGGGAGAAAAAUCGUCUUGCUAUUCAAAAUUGUAUUGUAAGGGGAUUCCUCGCCUAUGGAUCCUCUCGAGAUCCCUAACCACCAAAUCAGCCCU	CCGAGGGGAUCCAUGGGAGAA	CGCCUAUGGAUCCUCUCGAGA
Novel15	3	6928564	6928702	-	AAAGCUGACAGAAGAGAGUGAGCACACAUGGUGCCUUUCUUGCAUGAUGUAUGAUCGAGAGAGUUCAUGCUCGAAGCUAUGCGUGCUCACUUCUCUCUCUGUCAGCC	UGACAGAAGAGAGUGAGCAC	GCUCACUUCUCUCUCUGUCAGC
Novel16	4	138150590	138150710	+	UUGUUUGGGAGCAAGGAUACCAGAGAGGAUUGAAGGGGUUACAAUCUCUUUUCUAUUCAAAACUAAAUAGCAAGGGGAUUGUAGCCCCUCCAAUCCUCUCUGG	AGAGAGGAUUGAAGGGGUUAC	AGCCCCUCCAAUCCUCUCUGG

ID	chromosome	start	end	strand	Precursor_seqence	miRNA_seqence	miRNA*_seqence
Novel17	5	220222145	220222259	-	CUUCCACAGCUUUCUUGAACUGCAUCAUGCAUGCAGCAGGCUGUGCUGUGGACCUGAUCGAGUUUCAAUUGAUCCAAGCAAGCAAGAGGGCAGUUCAAUAAAGCUGUGGGAAAUU	GUUCAAUAAAGCUGUGGGAAA	UUCCACAGCUUUCUUGAACUG
Novel18	5	46506997	46507215	-	AUCCUAAUUCCUUCUAUGUCACUCGGAUGCAAUUUUUUACCCUUCAAUGCCAUCGCCAGCGGCAUAGUAGGUAUUAGGACAUGCAAUUUUCUUACCCUUUAUGCCACUGGCAAUGACAUGGAAGGGGAAGCAAUUGUGUCCGAGUGGCAUAGAAGGGAUUAGGAC	UUCAAUGCCAUCGCCAGCGGC	CACUGGCAAUGACAUGGAAGG
Novel19	6	34769920	34769994	-	CCCGCCGGCGAGCGCUUUCCUGCGAGGGCGCCGCCGUCGGUGA	GCGAGGGCGCCGCCGUCGGUGA	
Novel20	6	34951470	34951544	-	CCCGCCGGCGAGCGCUUUCCUGCGAGGGCGCCGCCGUCGGUGAGA	GCGAGGGCGCCGCCGUCGGUGA	
Novel21	6	7404512	7404639	+	UUGGCACGACAAAAAACUCGCACCGGGUGCUCUAAUCGGCGGUCGAUUACAGCGUCCAGUGCGAGUUCUUUGCCGUGCCAAAAUUGAAAGGAGGUCGGCUCGGAUGCCGGGGCAUGCAUACAGGACC	UGGCACGACAAAAAACUCGCA	CGAGUUCUUUGCCGUGCCAAAA
Novel22	6	91184717	91184896	+	AACUCAUCCGUGCACAUAUGCACAUAUUAAAUCUGGACCCUUCAUCUAAUAAUCAGCGGCUGAACUUGCACCUGAGUGUGGGGAGUGGAGUGCCAAAGGGUUUUUCUGUAAAUAUACCAGCCAUUGAUUGUUGGACGAAGGGUCUAGAUUUAAUAUGUGCAUAUAUACACGGAUGGGUC	UUAAAUCUGGACCCUUCAUCU	ACGAAGGGUCUAGAUUUAAUA
Novel23	7	153554237	153554455	-	AACCCGGAGGGAAUUGGAGGGGCUAAAAUCCCCUCCUUAUUCAAUUUUGAAUAAGAGGUGGAUUUUAUCCCCUUCAAUUCCCUCUGGUUUUGUGUCUCCCAAACUAGCCCUUAACAUUGGAAACAAGACAUGUUUAGAAUAACCGGGAAUAGAAACUCGAGCGAGAUAAAAGGGUGUACAAAAAUGAACGGGAAUAUUAAGCGGAAACUCAUAAUUUAA	CGGAGGGAAUUGGAGGGGCUA	UCCCCUUCAAUUCCCUCUGGU
Novel24	7	25824509	25824665	-	ACUUGCACACAUAGUCCCGAAUUGUGGAGAGAUUUUGAUAUUUAGGACUCAAUUUGCAUGCCUUUCGAGAUUUGACACCCCUAUCACAGUGACAUGUGGGGUCAUUGACAUGUGGCCAGAGUGUCAAAUCUUGAAAGGCAUGCGAAUUGGGUCCCAA	AGGCAUGCGAAUUGGGUCCCA	GGACUCAAUUUGCAUGCCUUU

ID	chromosome	start	end	strand	Precursor_seqence	miRNA_seqence	miRNA*_seqence
Novel25	7	5206366	5206460	-	AUGCCUGGCUCCCUGUAUGCCACUCGCACGGCUACCACACCCCCGGUGGCCGCUGCGGCUGGCGUGCGAGGUGCCAAGCAUGG	UGCCUGGCUCCCUGUAUGCCA	GCGUGCGAGGUGCCAAGCAUG
Novel26	8	105396488	105396571	-	GUCCAAGUCAAUGCAUCAUUGCCCCUAGUUUUGCCAUCAAAGAAGGCGAAGGCAGGGGCAAUGGUGCAUUGACUUGGUCAAAU	AUGGUGCAUUGACUUGGUCAA	GUCCAAGUCAAUGCAUCAUUG
Novel27	8	110490994	110491210	-	UGGCCCUUAGAUCUGACACGGUAUGCGGGUCAGCAGCCAGCAUGGCAUGCUUGCCAAGUUAGAUCUAAGGGCCAAACAAAGGGGU<br>AUGGACCUGUGGUGAACCACUUAAAAGUUUUUGGACCCGGAAGUUGAAUCUAAGGGCCAAAUGAAAGGGUAUGGACUUGCGGUGA<br>ACCACU	UGGCCCUUAGAUCUGACACGG	AAGUUAGAUCUAAGGGCCAAA
Novel28	8	174852885	174853005	-	UCCAUUUUAGUCCAUAAAUUGCCAAACACAGGGACUAAAAGUACUAAAACCUUGUUUUUAGUCCCUUUGUUUGGCAAUUUAAGGA<br>CUAAAAAAGUAAAAAUUAGUCCCUAGAAACCAAACG	UUUGGCAAUUUAAGGACUAAA	UAGUCCAUAAAUUGCCAAACA
Novel29	8	4080515	4080718	+	CACAAGAAUGGAGAUGUGAGGAUCUUUUUAAAAAUAUCCAGUGCGAGGUGGAAGGGUUGAUUUGGAAUAUGAUAUUUUUAAAAAG<br>GUCCUCCCACCUCCAUUCUUGGAUUGGUUUGCACAGUUUACACCUAAAAUGUUUCAUACUACAUAGGGCCAAUAAUCUUAUUACU<br>GUAUUGUGGGAUGAGUGGUGGGAGGUGG	AAGAAUGGAGAUGUGAGGAUC	UCCUCCCACCUCCAUUCUUGG
Novel30	8	46527943	46528072	-	AGGUUGUGAAGGCUAAGAAGUGUUUGUUUGGGAUUAUAAUCUACUCAGAUUAUGUAAUCCAACUUAUUUUGAAUUAAGUGCAACU<br>UUAAAAUAAGUUGGAUCAUAUAAUCUAG	AAGUUGGAUCAUAUAAUCUAG	AGAUUAUGUAAUCCAACUUAU
Novel31	8	62820042	62820127	-	GGAGGGAUUGGAGGGGCUAAAAUCCCCUCCUUAUUCAAAAUUGAAUAAGGAGGGGAUUUUAGCCCCUUCAUUCCCCUCCGGUU	AGCCCCUUCAUUCCCCUCCGG	GGAGGGAUUGGAGGGGCUAA
Novel32	8	75383833	75383955	+	CUCGGAGGGGAUUGAAGGGGCUAAAAUCCUCUUAUUCAAAUUUGAAUACGGAGUGCAUUUUAGCCUCUCCAAUCCCCUCUGGAUU	CUCGGAGGGGAUUGAAGGGGC	CUCUCCAAUCCCCUCUGGAUU
Novel33	8	96093889	96094099	+	UUGGUCCGUUAGAUUUAGAUCUAACCGUAGAUGCGAUCAUGGUUUGUUAGGAGCUUUUUUUAUAAAGCUACACGAGGUGGUGGUG<br>GAAGGGUUUAAAUGCUAAAUAUGACAUACGGUUGGAUCUAGAUCUAACGGACCAAGAGGCCUGCUUGCACGCUUACUUAAUUACU<br>GCUUGCAUAGUAGUAGUUGCCU	UUGGUCCGUUAGAUUUAGAUC	UCUAGAUCUAACGGACCAAGA

ID	chromosome	start	end	strand	Precursor_seqence	miRNA_seqence	miRNA*_seqence
Novel134	9	1345048	1345229	-	UCAUCCAGCGUAUCCAAUCUGAAGUCCCAGGCGAGAGGUGAUUAUCUUCCUAUCGCUCUAUGGCUGAUUCAAGCCCACUUAGAUUUGAUCCAAUUGAAAGCAAAGGUGACUGAGAAGAUGAGGGGAUUAUCACCUCUUACUGGGCCAUUCAGACUGGAUCACAUGGAUGGGA	AUCUGAAGUCCCAGGCGAGAG	CUUACUGGGCCAUUCAGACUG
Novel135	9	35218864	35218964	-	AUUGUAAGUCGUUCUAGCUUUUCUAAAUACAUAGAUUUUGCUACAUGCCUAGUAAAGCUAUGUAUCUUGAAAAGCUAGAACGAUUUACAAUUUGGA	UGAAAAGCUAGAACGAUUUACA	UAAGUCGUUCUAGCUUUUCUAA
Novel136	10	15564152	15564296	+	AGAUUGUAGGGGCUAGAAUCCCCUUCUUAUUCAAAAUUGAAUAAGAAAGGGAUUCUAGCCCCUCCAAUCCCCUCCGGAUUUGUGGCUCCC	AGAUUGUAGGGGCUAGAAUCC	AUUCUAGCCCCUCCAAUCCCC
Novel137	1	246675660	246675752	-	GUGGAUUGAGGGGGAUUGGAGAGGAUUAAAUCCUCUCCCAUACAAAUUUAUAUAGGAGGGGAUAUAAUUCCUUCCAAUUCCCUCAAUCCACCU		UUCCUUCCAAUUCCCUCAAUCC
Novel138	1	68549295	68549369	-	UCGGUUACCGGCCCUGAGCCAAGCUUAUAGCCUGUGGACCGACGACU	GCUUAUAGCCUGUGGACCGACG	UCGGUUACCGGCCCUGAGCCA
Novel139	2	192385214	192385333	+	UGGGACCAAGUGAAAUGGAGAGAAUUGAGGGGACUAUAAUCCCUUGUUAUUUAAAAUUGAAUAGCAAGGGAUUAUAGCCCCCUCAAUUCUCUUUGUUCCACUUACUCCCAAACAAGCCCU	GAAUUGAGGGGACUAUAAUCC	AUUAUAGCCCCCUCAAUUCUC
Novel140	2	41027838	41027974	+	GGAGUAGGUGGAACAAAGAGAAUUGAGGGGACUAUAAUCCCUUGCUAUUCAAUUUUGAAUAACAAGGGAUUAUAGCCCCCUCAAUUCUCUUUGUUUCACUUGGUCCCAAACAAGCCCUAAGUAGU	GAAUUGAGGGGACUAUAAUCC	AUUAUAGCCCCCUCAAUUCUC
Novel141	3	133857695	133857933	+	GAAGACGAGAUCUGGCCAGCCUGUGAUGAGAUACCGGAUCGAGCAUCUCUUGGAGACGGCACUGAGCAAAUAACACGAUGCCACCGUCAUCUUGGCAGAUUUUGUAGUUCCGCUGUCCAAUCGAUUUGUUGGUUUUAACUGACUUUGUCACCUUUUUAGUUUGCAAAAAUGGACAAUCUAAGUGACAAAGCCAGUUAAAACCAACAAAUUGGUUGGGCUGCGGAACUACAAA	UGUAGUUCCGCUGUCCAAUCG	GUUGGGCUGCGGAACUACAAA

ID	chromosome	start	end	strand	Precursor_seqence	miRNA_seqence	miRNA*_seqence
Novel42	3	174892164	174892459	-	CUCAAUCCGGACGCCGAUGUGGCGUGCCACGUUGGAGCAUACUAAUGAAAGUUCCGAGACCCAGUGACACAACAUGUCAAGUUUGGGACCCGAGUGACACAACAUGCCAAGUUCGAGGACCCAAGUGACUCUAACGUGGUACGCCACGUCGGUAUCCGGUUUGAGCAAAACCGGUUUUGUAUAGUUUUGCACGCGCCGACAAAGUUAGAUGACCCGUUUUUGCUGGUUUUCUAAGUUUGGUGACUGAAGUGACACAACGUGCCAAGUUCGAGGACCGAUACUGUAUUGUACUCAAA	UCAAUCCGGACGCCGAUGUGG	ACGUCGGUAUCCGGUUUGAGC
Novel43	3	197784121	197784247	+	UUGUUUGAGAGCCAAGGGAAUGGAGGGAAUUGGAGGGGUUAAAAUCCCCUUGUUAUUCAAAAUUGAAUAGGAAGGCGAUUUUAGCCCCUCUAAUCCACUUCAUUCCCCUUGCUUCCAAACAUGUCCU	UUGUUUGAGAGCCAAGGGAAU	UCCCCUUGCUUCCAAACAUGU
Novel44	4	28394972	28395101	+	UCCGGAGAGGAUUAGAGGGACUAAAAUCCCCGUUUUAUUUAAAAUUGAAUAAGGAGGAGAUUUUAGUCUCUCUAAUUCCCUCGGGUUUUGUGUCUCCCAAACUAGCCCUU	UCCGGAGAGGAUUAGAGGGACU	UCUCUCUAAUUCCCUCGGGUU
Novel45	6	171571090	171571198	-	CUAAAGAUCCAAAUAGGAAGCACUAAAACUGAUUAUAGUGGUGAAAAAAGUAUCAAUUUAGUCACUUUUAGCUCUUCCUGUUUGGAUAUUUAGCUCC	UUCCUGUUUGGAUAUUUAGCU	CUAAAGAUCCAAAUAGGAAGC
Novel46	8	125530731	125530942	-	AUAUUUGUUCGGGUCUCUAUCAGGUCCUAACGGGUUGUUAUGGCCCACAUGUUAGUCGUCGAGGAAGGAGAUAGAAGGGUGGGUGGGAUCCAACAGACAACGGCAACUGAUAUGUGGGACACAACAAUCCGUUAGGACGUAAUAGAGACCCGAACAAAGAUUGGGUCCUUAAAAGACAUACUUUAAAAGUUUAUGGACCGUGUUGACACA	GACACAACAAUCCGUUAGGAC	CCUAACGGGUUGUUAUGGCCC
Novel47	9	139046330	139046415	-	GUUGAAACCCAGCAAUCGAACAUAUAUUUUGUUGUAUAUCUGGUAAUACUUCAGAAUAUAUGUUCAAUUGUUGGGUUUCAACUU	UUCAAUUGUUGGGUUUCAACU	UUGAAACCCAGCAAUCGAACA
Novel48	4	10215889	10216062	+	AGAAUGUUUGCAGUCCCUCACAAAAAGGAAGAUAGACCCACCCAUAGCAGCAAUAACACAACUAAAACAUUAUCUGAUGGACCUACAGGUAAGGUCUAUAUUCUUUUUUGUGGGGGGCUGCAAACAUUCUGG	GGGGGGCUGCAAACAUUCUGG	AGAAUGUUUGCAGUCCCUCAC

ID	chromosome	start	end	strand	Precursor_seqence	miRNA_seqence	miRNA*_seqence
Novel49	3	216140788	216140927	+	UUAAGGCCUUGUUCGGUUGUGCCUGGAUCGAAUGAGAUUGGGGGGAUUAAAUCUUCUUCUAUUCAAUUUUGACUAGGAAGAGAUU UAAUCUCCUCCAAUCCCCUUCGAUCCAGACGUAACCGAACAAGCCCU	UCCAGACGUAACCGAACAAGC	UUGUUCGGUUGUGCCUGGAUC

Table S2. The m6A modification in the upstream or downsteam 1 Kb of the miRNA precursors.

miRNA Precursor (±1 Kb)	WW_fold_enrichment	WS_fold_enrichment	WW_location	WS_location
zma-MIR171k		11.18145		chr10: 138535210-138535901
zma-MIR171h	18.31629	24.88063	chr2: 18025427-18026191	chr2: 18025474-18026204
zma-MIR397b		3.08616		chr5: 84611244-84611443
zma-MIR167h	9.71453	5.94429	chr6: 95986825-95987245	chr6: 95986795-95987233
zma-MIR156j	5.25355	5.85345	chr7: 134662891-134663096	chr7: 134662821-134663020
zma-MIR319d	5.47325	4.70044	chr8: 27607568-27607791	chr8: 27607550-27607771
zma-MIR408b	18.0161	20.98938	chr8: 39584967-39585399	chr8: 39584978-39585425
zma-MIR169j	3.61262		chr2: 198436951-198437178	
zma-MIR164a	10.96522		chr2: 223402053-223402359	

Table S3. The detail of miRNA/target pairs for qRT-PCR verification.

miRNA	Target	p.value	Estimate
zma-miR166d-3p	Zm00001d048527	3.596e-05	-0.3735
zma-miR159a-3p	Zm00001d053589	4.062e-05	-0.3651
zma-miR319b-3p	Zm00001d053545	1.560e-05	-0.6979
Novel-31*	Zm00001d003518	3.469e-06	-0.5013

Additional file 1 Table S1. The detail of the novel miRNAs. miRNA stands for the miRNAs came from 5'-end of hairpin molecule, and miRNA* stands for the miRNAs came from 3'-end of hairpin molecule. **Table S2.** The m6A modification in the upstream or downsteam 1 Kb of the miRNA precursors. **Table S3.** The detail of miRNA/target pairs for qRT-PCR verification.

Figure S1

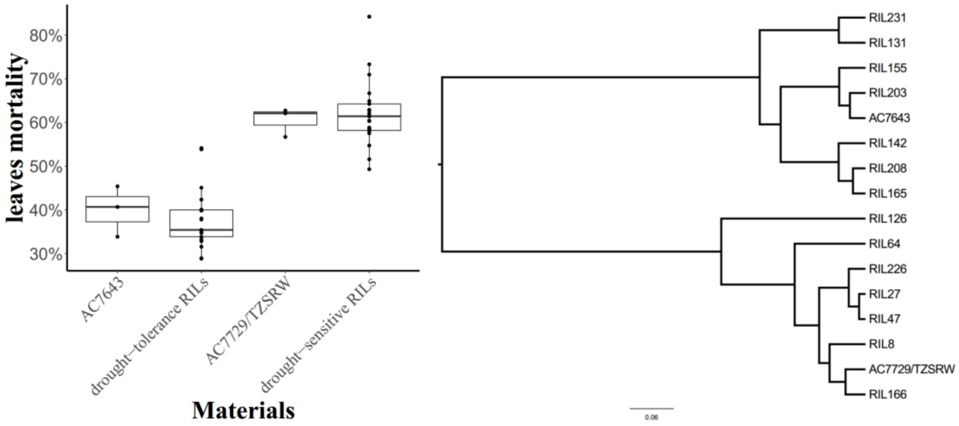

Additional file 2 Figure S1. The classification of RILs by leaf mortality.
